# Genetics, Screening, and Treatment of Familial Hypercholesterolemia: Experience Gained From the Implementation of the Vietnam Familial Hypercholesterolemia Registry

**DOI:** 10.3389/fgene.2020.00914

**Published:** 2020-08-14

**Authors:** Thanh-Huong Truong, Doan-Loi Do, Ngoc-Thanh Kim, Mai-Ngoc Thi Nguyen, Thanh-Tung Le, Hong-An Le

**Affiliations:** ^1^Vietnam National Heart Institute, Bach Mai Hospital, Hanoi, Vietnam; ^2^Department of Cardiology, Hanoi Medical University, Hanoi, Vietnam; ^3^School of Medicine and Pharmacy, Vietnam National University, Hanoi, Vietnam

**Keywords:** familial hypercholesterolemia, VINAFH Registry, low- and middle-income country, genetics, screening, treatment

## Abstract

Familial hypercholesterolemia (FH) is underdiagnosed and undertreated in a majority of the low- and middle-income countries. FH registries could prove useful in bridging the knowledge gaps, supporting genetic and clinical research, and improving health-care planning and patient care. Here, we report the first usage experience of the Vietnam FH (VINAFH) Registry. The VINAFH Registry was established in 2016 as a long-term database for prospective cohorts. FH patients were detected based on the opportunistic and cascade screening. Diagnosis of FH was assessed using the Dutch Lipid Clinic Network criteria, plasma levels of low-density lipoprotein (LDL) cholesterol, and genetic testing. To date, a total of 130 patients with FH have been registered, with 48 index cases and 82 relatives. Of the 130 patients, 8 were homozygous FH patients and 38 were children. Of FH individuals, 46.7% was confirmed by genetic testing: 61 patients (96.8%) carried the *LDLR* mutation (c.681C > G, c.1427C > G, c.1187-?_2140 ± ?del, c.2529_2530delinsA), and two patients (3.2%) carried the *PCSK9* (protein convertase subtilisin/kexin type 9) mutation (c.42_43insTG). The c.2529_2530delinsA mutation detected in this study is novel and reported only in the Vietnamese population. However, only 53.8% of FH patients were followed up post diagnosis, and only 15.3% of these were approved for lipid-lowering therapy and specialized care. Notably, factors such as knowledge about FH in patients and/or guardians of FH children and support of primary care physicians affected patient participation with respect to treatment strategies and follow-up. Genetic identification, screening, and treatment of FH were feasible in Vietnam. The VINAFH Registry significantly contributed to the formation of the government agencies legislative acts that established the importance of FH as a socially and medically important disease requiring appropriate management strategies. Other low- and middle-income countries could, thus, use the VINAFH Registry model as a reference to establish programs for FH management according to the current status.

## Introduction

Familial hypercholesterolemia (FH) is a common inherited disorder, affecting one in 250 individuals (de Ferranti et al., 2016; Akioyamen et al., 2017). This disease is characterized by elevated plasma levels of low-density lipoprotein cholesterol (LDL-C) that facilitates the development of atherosclerosis, premature coronary artery disease (CAD), and mortality (Neil et al., 2004; Cuchel et al., 2014). FH involves mutations in the gene encoding LDL receptor (*LDLR*; 90% of reported FH–causing variants), gene encoding apo-lipoprotein B (*APOB*; 5–10%), and, rarely, gene encoding protein convertase subtilisin/kexin type 9 (*PCSK9*; 1%) (Sturm et al., 2018).

Early identification and lipid-lowering therapy play important roles in reducing the health burden for FH. Screening programs such as cascade screening, universal screening, and opportunistic screening are effective for early detection of FH (Kirke et al., 2012). Previously, the Dutch Lipid Clinic Network (DLCN) score was widely accepted for FH diagnosis (Vallejo-Vaz et al., 2018). The European Society of Cardiology (ESC) and the European Atherosclerosis Society (EAS) recommend that lipid-lowering therapy should be started early and used optimally to achieve LDL-C goals (Mach et al., 2020). However, in reality, majority of FH patients remain underdiagnosed or undertreated. In most countries, less than 1% of FH patients are diagnosed (Nordestgaard et al., 2013). Furthermore, treatment for FH is still limited; for example, the CASCADE-FH Registry reported that only 48% of FH patients achieved LDL-C < 100 mg/dl and 22% achieved LDL-C < 70 mg/dl (Duell et al., 2019).

Because of these challenges, the EAS Familial Hypercholesterolemia Studies Collaboration (EAS-FHSC), the Familial Hypercholesterolemia Foundation, and the World Heart Federation initiated a global call to action for reducing the burden of disease and death due to FH (Vallejo-Vaz et al., 2015; Wilemon et al., 2020). Special attention was given to data registry as an important tool for providing knowledge and support management for FH. In fact, many developed countries have established FH registry. However, only a few low- and middle-income countries have established the same (Bamimore et al., 2015; Mehta et al., 2016; Vallejo-Vaz et al., 2018; Chen et al., 2019). Notably, Vietnam, a large and densely populated country in the Southeast Asia, has spurred rapid economic growth, greater than that of other low- and middle-income countries in the past years. With economic development, Vietnam has also achieved improvement in public health. However, changing lifestyles accompanying the economic growth lead to a double burden of disease; the burden of communicable disease remains, while the burden of non-communicable diseases, such as cardiovascular disease, is increasing (Nguyen and Hoang, 2018). Additionally, there exists a gap in the knowledge about cardiovascular disease, particularly FH. With a population at 97 million, we estimated 500,000 Vietnamese patients with FH, but most of them are underdiagnosed and undertreated (Vallejo-Vaz et al., 2018). This report describes the initial genetic characteristics, clinical characteristics, screening, diagnosis, and treatment of FH patients in Vietnam. We report the experiences gained from the implementation of the Vietnam Familial Hypercholesterolemia (VINAFH) Registry.

## Materials and Methods

**FIGURE 1:**
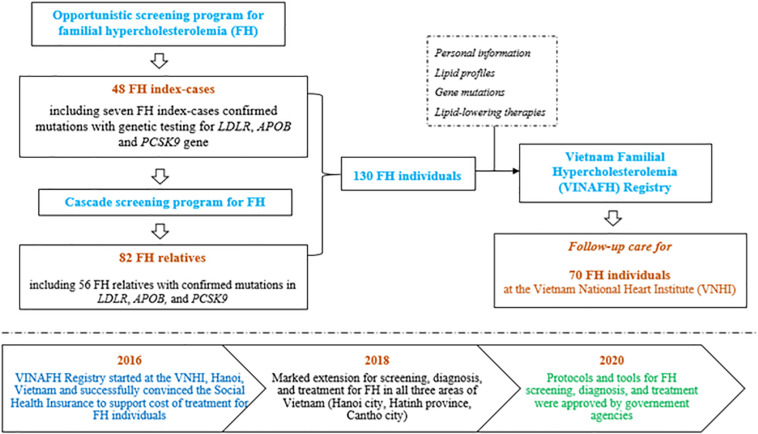
Flow diagram of implementation strategy of the Vietnam Familial Hypercholesterolemia (VINAFH) Registry.

On the basis of the success of our previous preliminary small-scope research on FH (Truong et al., 2018), we extended the study and established the VINAFH Registry as a long-term prospective cohort promoted by the Vietnam National Heart Institute (VNHI), Bach Mai Hospital – the largest hospital for cardiovascular disease in North Vietnam – and the national referral cardiovascular hospital. Implementation of the VINAFH Registry is presented in Figure 1. The VINAFH Registry was approved by the Council for Science of the VNHI, Bach Mai Hospital (No. 183/VTM-BVBM) and the Council for Science of the Ministry of Science and Technology of Vietnam (No. 828/GXNDGTD-BKHCN). The VINAFH Registry has the following three specific objectives: (1) to identify and enroll heterozygous and homozygous FH individuals in Vietnam; (2) to understand the clinical and genetic characteristics of FH individuals in Vietnam; and (3) to improve the management strategies for FH in Vietnam. Recruitment of FH individuals began in 2016 and is still ongoing. All the FH individuals included in this study provided informed consent. FH individuals younger than 18 years were enrolled only with the explicit consent of a parent or legal guardian.

### Inclusion Criteria for Familial Hypercholesterolemia Individuals in the Vietnam Familial Hypercholesterolemia Registry

We enrolled phenotypic and/or genotypic FH individuals, including index cases and their relatives in the Registry. Index case was detected by opportunistic screening in patients with premature CAD and/or hypercholesterolemia. Cascade screening was performed to detect FH relatives of index case, as previously described (Truong et al., 2018).

#### Phenotypic Familial Hypercholesterolemia Individuals

In adults, phenotypic FH was confirmed based on the DLCN criteria (WHO Human Genetics Programme, 1998). Patients with DLCN scores of 3–5 (possible FH), 6–8 (probable FH), and >8 (definite FH) were enrolled. In case of children < 18 years of age, phenotypic FH was diagnosed based on the presence of an LDL-C level consistent with FH in addition to a family history of premature CAD, and/or baseline high cholesterol in one parent, and/or presence of an FH-causing mutation (Wiegman et al., 2015). We also enrolled the likely FH phenotype relatives based on age- and gender-specific LDL-C cutoffs as described by Starr et al. (2008).

#### Genotypic Familial Hypercholesterolemia Individuals

Mutations in index cases were detected by massively parallel sequencing of FH genes (*LDLR*, *APOB*, and *PCSK9*) and multiplex ligation-dependent probe amplification (MLPA) of *LDLR*, as previously described (Hooper et al., 2012). Depending on the mutation present in the index case, genetic testing for relatives was performed by Sanger sequencing of the exon containing the family mutation, or by MLPA of *LDLR*. The ClinVar database, PolyPhen2, and MutationTaster were used to confirm the mutations. Genotypic FH individuals were classified as homozygous for FH (HoFH), carrying the same mutations in both alleles of FH genes, and heterozygous for FH (HeFH), carrying only one mutation in the alleles of FH genes.

### Exclusion Criteria for Familial Hypercholesterolemia Individuals in the Vietnam Familial Hypercholesterolemia Registry

Individuals with known medical conditions other than FH that contribute to hyperlipidemia, such as hypothyroidism, nephrotic syndrome, cholestasis, and hypopituitarism, were excluded from the VINAFH Registry. Medical, family, and clinical history were recorded for all registered individuals: characteristics based on the DLCN criteria (WHO Human Genetics Programme, 1998), lipid profiles, and mutation characteristics. Furthermore, information regarding risk factors for cardiovascular disease, including age, sex, smoking, diabetes, hypertension, obesity, premature CAD, and ongoing/past lipid-lowering therapies, were also collected in this Registry.

### Outcome Measures

Primary outcomes:

– Number of FH individuals identified.
– Genetic testing for FH-causing mutations (*LDLR*, *PCSK9*, and *APOB*).

Secondary outcomes:

– Number of FH individuals who received dietary and lipid-lowering therapy.
– Number of FH individuals who achieved LDL-C targets.
– Incidence of cardiovascular events (coronary, cerebral, or peripheral vascular diseases) and cardiovascular cause detected during annual follow-up.
– Contribution of the Registry in the formation of national health policies concerning the recognition of FH as an important disease with development of appropriate management strategies.

### Statistical Analyses

Continuous variables are expressed as the arithmetic mean and standard deviation if normally distributed and as the median with inter-quartile range for non-normal distribution. Categorical variables are expressed as count and percentage. Normally distributed continuous variables between two groups were compared using the Student *t*-test for independent samples. Non-normally distributed continuous variables were compared using the Mann–Whitney *U* test. Proportions were compared by using the chi-squared test with continuity correction or Fisher’s exact test, when appropriate. The analyses were performed with IBM SPSS 22.0 software. The results with *p* values below 0.05 were considered statistically significant.

## Results

### Genetic Identification of Familial Hypercholesterolemia Individuals

A total of 130 FH individuals have been registered. Of these, 63 FH individuals (48.5%) underwent genetic testing, confirming the presence of mutations in the genes studied (*LDLR*, *APOB*, and *PCSK9*); eight individuals were HoFH and 55 were HeFH. Four *LDLR* mutations and one *PCSK9* mutation were identified in these individuals, while no pathogenic variants of *APOB* were identified. Mutations in *LDLR* were identified in 6/7 index cases (85.7%) in this Registry. We found novel mutations in *LDLR*, which has not been reported but are annotated in the ClinVar database: c.2529_2530delinsA, (p.Asp843Glufs*86). The variants and their distribution in the study cohort are given in Table 1.

**TABLE 1:** *LDLR* and *PCSK9* variants identified in the VINAFH Registry.

Gene	Exon	Variant	Predicted effect	Number of index case (*n* = 7)	Number of relative (*n* = 56)	Number of HoFH (*n* = 8)	Number of HeFH (*n* = 55)
*LDLR*	E.4	c.681C > G	p.Asp227Glu	2 (28.6%)	16 (28.5%)	2 (25%)	16 (29.1%)
*LDLR*	E.10	c.1427C > G	p.Pro476Arg	2 (28.6%)	11 (19.6%)	2 (25%)	11 (20%)
*LDLR*	E.9–E.14	c.1187-?_2140 ± ?del	–	1 (14.3%)	20 (35.7%)	4 (50%)	17 (30.1%)
*LDLR^∗^*	E.17	c.2529_2530delinsA	p.Asp843Glufs^∗^86	1 (14.3%)	8 (14.3%)	0	9 (16.4%)
*PCSK9*	-	c.42_43insTG	p.Leu15fs	1 (14.3%)	1 (1.8%)	0	2 (3.6%)

*LDLR, low-density lipoprotein receptor; PCSK9, proprotein convertase subtilisin/kexin type 9; FH, familial hypercholesterolemia; HoFH, homozygous familial hypercholesterolemia; HeFH, heterozygous familial hypercholesterolemia; VINAFH, Vietnam Familial Hypercholesterolemia. ^∗^Novel mutation that has not previously been reported and is absent from the ClinVar database.*

### Screening of Familial Hypercholesterolemia Individuals

We identified 48 FH index cases and 82 FH relatives. Clinical characteristics of FH individuals are given in Table 2. Notably, the mean ± standard deviation of age for FH diagnosis was 34.8 ± 1.95 years, with an earlier age at diagnosis in the relative group compared with that in the index-case group (28.6 ± 2.43 vs. 45.3 ± 2.66 years, respectively, *p* < 0.001).

**TABLE 2:** Baseline characteristics of FH individuals in the VINAFH Registry.

Characteristics	Total (*n* = 130)	Index cases FH (*n* = 48)	Relatives FH (*n* = 82)	*p* value
Age, year	34.8 ± 1.95 (30.9–38.6)	45.3 ± 2.66 (40.0–50.7)	28.6 ± 2.43 (23.8–33.4)	<0.001
Children less than 18 years old, *n* (%)	41 (31.5%)	5 (10.4%)	36 (43.6%)	<0.001
Male, *n* (%)	73 (56.2%)	34 (70.8%)	39 (47.6%)	0.01
PCAD, *n* (%)	33 (25.4%)	31 (64.6%)	2 (2.4%)	<0.001
Smoking, *n* (%)	29 (22.3%)	20 (41.7%)	9 (11%)	<0.001
Hypertension, *n* (%)	20 (15.4%)	10 (20.8%)	10 (12.2%)	0.188
Diabetes, *n* (%)	2 (1.5%)	0 (0%)	2 (2.4%)	0.531
Obesity, *n* (%)	18 (13.8%)	8 (16.7%)	10 (12.2%)	0.476
Xanthomas, *n* (%)	23 (17.7%)	16 (33.3%)	7 (8.5%)	<0.001
Arcus cornealis, *n* (%)	17 (13.1%)	13 (27.1%)	4 (4.9%)	0.001
TC, mmol/L	8.43 ± 0.28 (7.88–8.98)	9.83 ± 0.56 (8.69–10.96)	7.6 ± 0.25 (7.11–8.09)	<0.001
LDL-C, mmol/L	6.48 ± 0.26 (5.96–7.0)	7.63 ± 0.55 (6.52–8.74)	5.8 ± 0.23 (5.34–6.25)	0.001
HDL-C, mmol/L	1.25 ± 0.28 (1.2–1.31)	1.21 ± 0.05 (1.11–1.31)	1.28 ± 0.03 (1.21–1.35)	0.24
Triglyceride, mmol/L	1.87 ± 0.12 (1.62–2.11)	2.23 ± 0.24 (1.74–2.71)	1.66 ± 0.13 (1.39–1.92)	0.027

*PCAD, premature coronary artery disease (≤55 years in men, ≤60 years in women); TC, total cholesterol; LDL-C, low-density lipoprotein cholesterol; HDL-C, high-density lipoprotein cholesterol; FH, familial hypercholesterolemia; VINAFH, Vietnam Familial Hypercholesterolemia.*

### Treatment of Familial Hypercholesterolemia Individuals

Post diagnosis, 53.8% (*n* = 70/130) of FH individuals continued to follow-up at the VNHI. All individuals were given standard treatment with dietary supplements containing plant stanols for controlling cardiovascular risks. Only 15.3% (*n* = 11/70) were given lipid-lowering therapy described in Table 3. Of these 11 individuals, five were HoFH while six were HeFH. After treatment with lipid-lowering therapies for 1 year, 83.3% (*n* = 5/6) of HeFH patients had LDL-C < 2.5 mmol/L, while the mean of plasma LDL-C of HoFH patients reduced from 17.5 ± 6.0 mmol/L at the time of diagnosis to 10.2 ± 4.1 mmol/L. No new cardiovascular events or mortalities were observed in these individuals. The lower incidence of lipid-lowering therapy was attributed to patient refusal. We recorded two important reasons for patient refusal to lipid-lowering therapy in case of individuals in the VINAFH Registry: lack of knowledge about the effects and side effects of lipid-lowering therapy and barriers due to treatment cost. Besides, FH individuals only agreed to lipid-lowering therapy after we contacted their primary care physicians and successfully convinced these physicians to join as collaborators in our network for FH management.

**TABLE 3:** Lipid-lowering therapy in 11 FH individuals.

Therapy	All (*n* = 11)	HoFH (*n* = 5)	HeFH (*n* = 6)
Statins (*n*,%)	11 (100%)	5 (100%)	6 (100%)
High-intensity statins^∗^ (*n*,%)	7 (63.6%)	5 (100%)	2 (33.3%)
Ezetimibe	4 (36.4%)	3 (60%)	1 (16.7%)
Plant stanols	3 (27.3%)	3 (60%)	0
Plasma exchange	2 (18.2%)	2 (40%)	0
Lipoprotein apheresis	0	0	0
PCSK9 inhibitors	0	0	0

*PCSK9, proprotein convertase subtilisin/kexin type 9; HoFH, homozygous familial hypercholesterolemia; HeFH, heterozygous familial hypercholesterolemia; FH, familial hypercholesterolemia. ^∗^ Atorvastatin 40 or 80 mg and rosuvastatin 20 mg were defined as high-intensity statins.*

### Impact of the Vietnam Familial Hypercholesterolemia Registry on Health Policy

On the basis of experiences gained from the implementation the VINAFH Registry, we created academic documents, including (1) screening and testing (diagnostic and genetic) protocols for FH; (2) guidance on genetic counseling for FH; (3) tools for FH screening, diagnosis, and treatment; and (4) management model for FH. All of them were evaluated, appraised, and approved for clinical practice in Vietnam by the Ministry of Science and Technology of Vietnam. Further, we successfully convinced the Social Health Insurance to cover treatment cost for FH individuals who participated in the VINAFH Registry and were followed up at the VNHI.

## Discussion

In our Registry, we registered a higher rate of FH individuals with confirmed mutations (48.5%, *n* = 63/130) than did few other countries (Nordestgaard and Benn, 2017), based on the support of national genetic experts and colleagues from Australia. We identified four different mutations in *LDLR* and one in *PCSK9*. The majority of FH individuals carried mutation in *LDLR* (85.7% of index cases and 96.8% in total), which is similar to that reported in previous studies (Soutar and Naoumova, 2007; Sjouke et al., 2015; Khera et al., 2016). We found that the exon mutations of *LDLR* were varied, which was also reported by a previous study in a Korean population (Lee, 2016). *LDLR* p.Asp227Glu (FH Afrikaner-1, FH Maine) missense variant, which occurs within repeat 5 of the ligand-binding domain of LDLR, has previously been identified (Kotze et al., 1993; Hooper et al., 2012; Sharifi et al., 2016). *LDLR* c.1187-?_2140 + ?del and *PCSK9* c.42_43insTG have also been identified in several cohorts of FH patients (Goldmann et al., 2010; Clinvar database, 2020). Notably, two *LDLR* mutations (p.Pro476Arg and p.Asp843Glufs*86) that we identified in Vietnamese patients have not been recorded in other ethnicities as confirmed by the Clinvar database (2020). We previously reported the identification of *LDLR* p.Pro476Arg missense variant, which occurs within the gene encoding the EGF spacer domain of the LDLR, in two Vietnamese families with two HoFH index cases and 11 HeFH relatives (Truong et al., 2018). Pro476 is conserved across species, and prediction algorithms PolyPhen2 and MutationTaster suggest that Pro476Arg is pathogenic. In the VINAFH Registry cohort, we identified the novel mutation, *LDLR* p.Asp843Glufs*86 frameshift variant, which occurs within the gene encoding the cytoplasmic domain of the LDLR; it was detected in a family with nine HeFH individuals. This frameshift variant results in the loss of 18 amino acids and addition of 86 amino acids at the C-terminal of the LDLR. In general, simultaneous occurrence of both the previously reported variants and the novel variant detected in this study suggest a broad spectrum of mutations and high heterogeneity of FH in the Vietnamese population, which is similar to that observed in other countries (Jiang et al., 2015; Fairoozy et al., 2017).

Massively parallel sequencing detects structural variations with high sensitivity and specificity. However, it is inefficient to detect large deletions/duplications. In contrast, MLPA is highly sensitive to detect these mutations. A previous report showed that in 19/377 (5%) patients with suspected FH, no mutation was found with massively parallel sequencing, whereas MLPA identified large deletions/duplications in *LDLR* (Taylor et al., 2009). Thus, besides combined massively parallel sequencing, the MLPA method is useful for genetic testing in FH patients. Interestingly, our first index case with HoFH phenotype was also tested using massively parallel sequencing with targeted analysis of FH genes, but no mutations were detected. Therefore, the MLPA method was used to confirm this index case, and homozygotes for a deletion of exons 9 to 14 of *LDLR* were found. We also detected three HoFH relatives and 17 HeFH relatives carrying this mutation (Truong et al., 2018).

Screening is the first step to improve status of underdiagnosed and undertreated FH cases. In the VINAFH Registry, we combined opportunistic and cascade screening to increase the likelihood of detection. Opportunistic screening was the approach used to detect FH index cases in high-risk individuals such as those with premature CAD, hypercholesterolemia, xanthomas, and arcus cornealis (Nordestgaard et al., 2013). This approach was selected based on a high prevalence of FH in patients with premature CAD (Nanchen et al., 2015; Faggiano et al., 2018; Pirazzi et al., 2019). Elevated plasma total cholesterol and LDL-C levels are important clinical signs of FH. A previous study reported that 1.7% of individuals with LDL-C ≥ 190 mg/dl carried FH mutation (Khera et al., 2016). Moreover, xanthomas and arcus cornealis are also key signs of FH, especially in homozygotes (Bujo et al., 2004). Compared with previous preliminary small-scope report, including the first five FH index cases in Vietnam (Truong et al., 2018), the VINAFH Registry has significantly expanded the number of FH individuals, including 48 index cases detected by opportunistic screening. This provided evidence for the effectiveness of opportunistic screening, especially in developing countries where FH is underdiagnosed.

Because FH is dominantly inherited, each new FH case could become an index case for cascade screening. Indeed, this is a highly effective method for detecting FH in family members and has been approved in many countries (Bell et al., 2015; Jannes et al., 2015; Rubio-Marin et al., 2018). In fact, we undertook cascade screening for FH in close relatives of index cases and detected 82 FH cases. On average, 14 new cases of FH were detected per HoFH index case (Truong et al., 2018). The VINAFH Registry revealed that FH relatives had a younger mean for age than FH index cases. Interestingly, 43.6% of FH relatives were children < 18 years old. Fortunately, only 2.4% of FH relatives had a history of premature CAD, which was lower than the FH index cases (64.6%). Early diagnosis and, thus, prevention or delaying the onset of atherosclerotic cardiovascular disease are the most important factors in the management of FH (Nordestgaard et al., 2013; Knowles et al., 2017; Mach et al., 2020).

The VINAFH Registry has also shown a high prevalence of FH individuals with cardiovascular risk factors including smoking, hypertension, and obesity. Like low- and middle-income countries, Vietnam also faces the increasing burden of non-communicable diseases (Bennett et al., 2018). In a national survey of risk factors for non-communicable diseases in 2015, prevalence of smoking, hypertension, and overweight/obesity was 55.7, 18.5, and 21.1%, respectively, in men and 1.73, 10.2, and 21.2%, respectively, in women (Bui et al., 2016). In FH management, controlling cardiovascular risk factors has been reported to be highly beneficial for reducing cardiovascular events and mortality (Akioyamen et al., 2019). Therefore, the prevention program for FH in Vietnam will focus on lifestyle modification education.

Considerable efforts have been taken for the management of FH in Vietnam, and 53.8% of FH individuals continued to follow-up post diagnosis. FH individuals were also educated in lifestyle modification. However, a large number of Vietnamese FH individuals were undertreated, which is also commonly observed in many countries (Nordestgaard et al., 2013). The VINAFH Registry showed that traditional lipid-lowering therapies, including statin, ezetimibe, plant stanols, and plasma exchange, were effective for reducing plasma LDL-C levels in FH individuals. As per the ESC/EAS guidelines, statin is recommended as the first-line therapy for FH, and high-intensity and maximal potent statin doses are preferred (Nordestgaard et al., 2013; Cuchel et al., 2014; Besseling et al., 2016; Mach et al., 2020). However, in the VINAFH Registry, prevalence of HeFH individuals prescribed with high-intensity statins was limited (33.3%), similar to previous reports in China (Auckle et al., 2017). In Japan, which is a developed country, only 19.2% of FH individuals were treated with high-intensity statins (Teramoto et al., 2018). Lipoprotein apheresis and PCSK9 inhibitors, which are currently presented as efficient methods of treatment of FH (Mach et al., 2020), are still not available in Vietnam. A previous survey by the EAS-FHSC showed that these lipid-lowering therapies are limited in most countries (Vallejo-Vaz et al., 2018).

Limited use of lipid-lowering therapies for FH individuals could be explained by the lack of knowledge and awareness about the disease. Our interviews with FH individuals or legal guardians of FH children noted confusion about the effects and side effects of long-term drug use. It should be noted that in the health-care system, patient-primary care physician relationship is of the utmost importance; everybody highly trusts their physician (O’Malley et al., 2004). Primary care physicians and cardiologists generally advice the patients regarding health and prescribe drugs on the basis of their knowledge. Therefore, updating the physicians’ knowledge about FH is significant to obtain consent for the patient’s treatment. However, Vietnamese physicians had a large deficit in FH knowledge and awareness (Pang et al., 2017). This emphasizes the critical importance of implementing education and awareness programs for both FH individuals and physicians. Ideally, physicians treating FH individual should collaborate for FH management.

As mentioned, many low- and middle-income countries suffer from a double disease burden, the backlog of common infections, and the emerging challenges of non-communicable diseases; moreover, their health resources are also limited. If the relevant information is not available to the government agencies, they may omit important diseases, such as FH, from the national health policy. Therefore, scientists play important roles for providing evidence accumulated though such registries and persuade government agencies to adjust the health policies. In our case, we conducted a preliminary small-scope research for FH, then extended it through the VINAFH Registry, and reported the updated results about FH status in Vietnamese individuals to the government agencies. Simultaneously, we convinced the Social Health Insurance to support cost of treatment for the registered FH individuals. In our experience, initial results of such a registry should provide data and documentation regarding evidence of the existence of the disease pathology, initial results of FH management, and protocols and tools for screening, diagnosis, and treatment of FH. Notably, including genetic information into the registry provides high-value scientific evidence that is a useful factor that increases the persuasiveness of the study for government agencies. Thus, the VINAFH Registry has significantly contributed to the formation of government agencies legislative acts, establishing FH as a socially and medically important disease with appropriate management strategies. It has also led to the deployment of a national screening and disease management program for FH in Vietnam in the future.

## Conclusion

In conclusion, the VINAFH Registry is the first database on genetic screening and management of FH in the Vietnamese population. Moreover, we reported a novel variant in *LDLR* that were identified in our cohort. The likely occurrence of a complex of FH mutations suggests the need for a national FH genetic study. Based on the findings of this study with respect to the treatment strategies for Vietnamese FH patients, we propose the need for awareness and educational programs about FH for patients and doctors, so as to increase the number of diagnosed and treated patients. The VINAFH Registry had an important contribution in the formation of government agencies legislative acts concerning the establishment of FH as a socially and medically important disease and development of appropriate management strategies. Low- and middle-income countries might refer to our Registry to establish similar programs for the management of FH on the basis of genetic testing combined with opportunistic and cascade screening. The management strategies for FH should be implemented in a step-by-step manner on the basis of the personal and financial resources available in these countries.

## Data Availability Statement

The original contributions presented in the study are included in the article/supplementary material, further inquiries can be directed to the corresponding authors.

## Ethics Statement

The studies involving human participants were reviewed and approved by the Council for Science of the VNHI, Bach Mai Hospital (No. 183/VTM-BVBM) and the Council for Science of the Ministry of Science and Technology of Vietnam (No. 828/GXNDGTD-BKHCN). Written informed consent to participate in this study was provided by the participants’ legal guardian/next of kin.

## Author Contributions

T-HT and D-LD initiated the study, designed data collection tools, monitored data collection, cleaned and analyzed the data, and drafted and revised the manuscript. N-TK and M-NN monitored data collection, cleaned and analyzed the data, and revised the draft manuscript. T-TL and H-AL monitored data collection and revised the draft manuscript. All authors read and approved the final manuscript.

## Conflict of Interest

The authors declare that the research was conducted in the absence of any commercial or financial relationships that could be construed as a potential conflict of interest.
